# Ion Channels: New Actors Playing in Chemotherapeutic Resistance

**DOI:** 10.3390/cancers11030376

**Published:** 2019-03-16

**Authors:** Philippe Kischel, Alban Girault, Lise Rodat-Despoix, Mohamed Chamlali, Silviya Radoslavova, Hiba Abou Daya, Thibaut Lefebvre, Arthur Foulon, Pierre Rybarczyk, Frédéric Hague, Isabelle Dhennin-Duthille, Mathieu Gautier, Halima Ouadid-Ahidouch

**Affiliations:** 1 Laboratoire de Physiologie Cellulaire et Moléculaire (EA 4667), Université de Picardie Jules Verne, UFR des Sciences, 33 Rue St Leu, 80039 Amiens, France; alban.girault@u-picardie.fr (A.G.); lise.despoix@u-picardie.fr (L.R.-D.); mohamed.chamlali@etud.u-picardie.fr (M.C.); silviya.radoslavova@u-picardie.fr (S.R.); hiba.abou.daya@etud.u-picardie.fr (H.A.D.); thibaut.lefebvre@u-picardie.fr (T.L.); arthfoulon@gmail.com (A.F.); rybarczykpierre@hotmail.com (P.R.); fh-lnc@u-picardie.fr (F.H.); isabelle.dhennin@u-picardie.fr (I.D.-D.); mathieu.gautier@u-picardie.fr (M.G.); halima.ahidouch-ouadid@u-picardie.fr (H.O.-A.); 2 Service de Gynécologie Obstétrique, CHU Amiens Picardie, 80000 Amiens, France; 3 Laboratoire d’Anatomie et Cytologie Pathologiques, CHU Amiens Picardie, 80000 Amiens, France

**Keywords:** ion channels, cancer, chemoresistance

## Abstract

In the battle against cancer cells, therapeutic modalities are drastically limited by intrinsic or acquired drug resistance. Resistance to therapy is not only common, but expected: if systemic agents used for cancer treatment are usually active at the beginning of therapy (i.e., 90% of primary breast cancers and 50% of metastases), about 30% of patients with early-stage breast cancer will have recurrent disease. Altered expression of ion channels is now considered as one of the hallmarks of cancer, and several ion channels have been linked to cancer cell resistance. While ion channels have been associated with cell death, apoptosis and even chemoresistance since the late 80s, the molecular mechanisms linking ion channel expression and/or function with chemotherapy have mostly emerged in the last ten years. In this review, we will highlight the relationships between ion channels and resistance to chemotherapy, with a special emphasis on the underlying molecular mechanisms.

## 1. Introduction

Cancers cells have the ability to develop resistance to traditional therapies. Drug resistance may be intrinsic (i.e., present before chemotherapy exposure), but tumor cells can also acquire drug resistance. Indeed, although systemic agents used for cancer treatment (cytotoxic, hormonal, and immunotherapeutic agents) are usually active at the beginning of therapy (90% of primary breast cancers and 50% of metastases for instance), about 30% of patients with early-stage breast cancer will have recurrent disease. Resistance to therapy is not only common, but expected [1]. Tumor cells from recurrent tumors exhibit increased resistance to chemotherapeutic drugs [2], and exposure to chemotherapeutic drugs can promote development of drug resistance in tumor cells, leading to subsequent failure of the chemotherapeutic treatment [3], thus drastically limiting effective therapeutic modalities. Residual tumor cells are detected post-treatment in most cancer patients, and these cells are thought to remain in a quiescent state for years before resuming growth, resulting in tumor recurrence. Deciphering molecular mechanisms of this acquired cellular resistance is therefore mandatory for predicting tumor resistance and to allow discovery of new treatments. Several mechanisms underlying cell resistance have been identified so far. Among those, membrane transporters and ion channels have been shown to play key roles in chemosensitivity. These include [4]:

- Decreased activity of the uptake transporters, or alternatively enhanced efflux for water-soluble drugs (such as cisplatin)
- Increased drug efflux mediated by energy-dependent transporters. For hydrophobic drugs (such as vinblastine, doxorubicin, and paclitaxel), entry occurs largely by diffusion across the membrane, although this process can also be critically enhanced by transport proteins
- Indirect mechanisms by which transporters and channels affect chemosensitivity by modulating apoptosis pathways or the efficiency of drug diffusion along electrochemical gradients into cells

This review will more specifically focus on ion channels. Altered expression of ion channels is now considered as one of the hallmarks of cancer [5], and several ion channels have been linked to cancer cell resistance. While some ion channels have been associated with chemoresistance since the 80s [6], extensive description of molecular mechanisms involved in chemoresistance is more recent (early 2000s [7], with the vast majority of scientific articles being published after 2010). In this review, we will highlight the relationships between channels (calcium, potassium, magnesium and chloride channels) and resistance to chemotherapy, with special emphasis on the underlying molecular mechanisms.

## 2. Calcium Channels

### 2.1. Plasma Membrane Ca^2+^ Channels

Calcium (Ca^2+^) is a well-known ubiquitous second messenger regulating a wide variety of physiological functions [8,9], including cell death [10]. Disruption of Ca^2+^ homeostasis was reported in many pathological conditions, including cancer [11,12]. There is a ~10,000-fold concentration gradient for Ca^2+^ across the plasma membrane, and three major classes of membrane-associated proteins directly involved in Ca^2+^ regulation: channels, pumps (ATP-ases), and exchangers. These proteins have different cellular and tissue distribution and their regulation occurs through multiple signalling pathways ([13] for review). Extracellular Ca^2+^ can enter cells by several classes of channels, including:

- Voltage-operated channels, activated by membrane depolarization
- Second messenger-operated channels, activated by small messenger molecules such as inositol phosphates, cyclic nucleotides, and lipid-derived messengers (diacyl-glycerol, arachidonic acid and its metabolites)
- Receptor-operated channels, activated by direct binding of a neurotransmitter or hormone agonist
- Store-operated channels (SOC), activated by depletion of intracellular Ca^2+^ stores [14]

SOC Entry (SOCE) is one of the main calcium entries in non-excitable cells, and typically allows calcium influx through the plasma membrane, subsequently to endoplasmic reticulum depletion. This ubiquitous SOCE pathway is not only necessary to refill internal calcium stores, but also to activate downstream signalling cascades [15,16]. Apoptosis is also potentially triggered when a large and sustained rise in cytosolic calcium occurs through SOCE (mediated by store-operated channels SOCs [17,18,19]). Actors of this mechanism include depletion sensors (STIM reticular proteins, the STIM1 and STIM2 isoforms), as well as plasma membrane channels. Among these, Orai channels represent highly selective calcium channels (with 3 distinct Orai isoforms described to date, Orai1, Orai2 and Orai3), while TRP (Transient Receptor Potential) channels are non-selective calcium channels.

Ca^2+^ entries can also occur without any stimulation, suggesting that some Ca^2+^ channels are constitutively active in resting conditions. Interestingly, these basal Ca^2+^ influxes are linked to malignant transformation, but the underlying molecular mechanisms are far from being understood ([20], for review).

In addition, under certain conditions, Ca^2+^ can enter cells via the Na^+^/Ca^2+^ exchanger (NCX) operating in reverse mode. Cytosolic calcium concentration can also rise following endoplasmic reticulum depletion (see Section 2.2. Intracellular Calcium Stores below).

Chemoresistance has been first associated with Ca^2+^ channel activity in the 80s. Blockade of Ca^2+^ channels was associated with the enhancement of anticancer drug cytotoxicity ([21], for review). It was notably demonstrated that combination of verapamil (a calcium channel blocker) with antineoplastic agents was able to potentiate the efficacy of chemotherapeutic agents in drug-sensitive malignancies, and was also able to confer chemosensitivity in resistant tumor cells [22]. However, there was inconclusive evidence for the role of Ca^2+^ ions and Ca^2+^ channels in the modulation of chemoresistance, and furthermore, there was no evidence that Ca^2+^ channels blocking activity per se was necessary for the reversal of drug resistance [21]. Although Ca^2+^ channels blockers have been studied in combination with chemotherapeutic drugs for several decades, it is noteworthy that the mechanism of action for these Ca^2+^ blockers in inhibiting the growth of some drug-resistant tumors was entirely independent of Ca^2+^ channels modulation, and instead appeared to be related to non-specific interactions with the MultiDrug Resistance-1 (MDR1) protein ([23] for review). For instance, Kiwit et al. have shown that calcium antagonists did not influence chemoresistance in gliomas that do not express MDR1. On the other hand, addition of calcium antagonists to the adjuvant chemotherapy could overcome primary chemoresistance only in tumors expressing the multidrug-resistant phenotype [24].

Since the beginning of the 2010s, a growing number of studies have shown a clear link between Ca^2+^ channels expression (and/or function) and sensitivity to chemotherapeutic drugs. Many calcium channels are involved in chemoresistance, against a number of chemotherapeutic drugs, in a large variety of cancers. We will more specifically focus on the main calcium entry, SOCE, and its key actors, the Orai and TRP channels.

#### 2.1.1. Orai/Stim Mediated SOCE and Chemoresistance

Depending on cancer cell lines and chemotherapeutic drugs, SOCE can lead to increased or decreased resistance. First, because each cell line differentially express Orai and Stim proteins. Second because both Orai1 and Stim1 were shown to regulate the activity of a number of intracellular effectors including PKC β2 [25], PKC δ [26], extracellular signal-related kinases ERK1/2 [27], and both cytoplasmic kinase Pyk2 and calpain [28]. Third, both Orai1 and Stim1 were shown to have SOCE independent functions. For instance, the Secretory Pathway Ca^2+^-ATPase SPCA2 can activate Orai1 independently of ER calcium stores or Stim proteins [29]. Within such a complex regulatory network and the role of Orai1/Stim1 downstream targets in both pro-survival and pro-apoptotic processes, the resistance of each cell line is likely to depend on the activated signalling pathway [30].

##### Increased Resistance Conferred by the Overexpression of Stim and Orai Proteins

**Figure 1:**
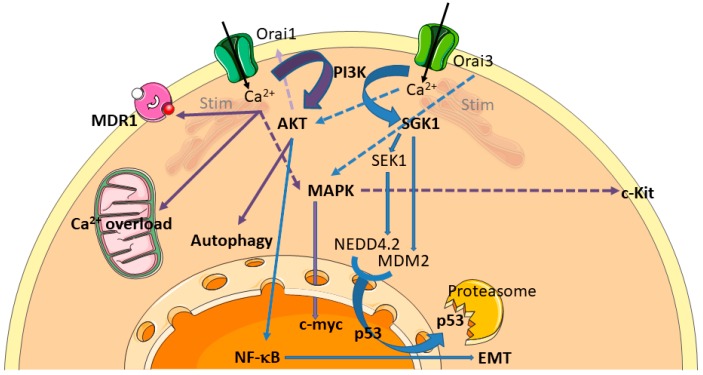
Chemoresistance pathways involving Orai calcium channels. Orai calcium channels are able to modulate sensitivity to chemotherapy. Processes known to alter chemoresistance are highlighted in bold. These include calcium overload, MultiDrug Resistance (MDR), autophagy, modulation of signalling pathways (MAPK and PI3K-Akt/Sgk), transcription factors (NF-κB, c-myc, p53), and EMT (Epithelial to Mesenchymal Transition). Processes initiated by Orai1 and Orai3 are summarized by purple and blue arrows, respectively. The dashed arrows are not different from plain arrows, except that they cross other arrows.

Numerous studies have now established a clear link between the Orai/Stim complex and cell resistance, although the mechanisms are not always accurately described. For instance, Stim1-mediated SOCE protects osteosarcoma cells from undergoing cisplatin-induced apoptosis. Knockdown of Stim1 effectively sensitizes cells to cisplatin via promoting Endoplasmic Reticulum (ER) stress-mediated apoptosis [31]. In pancreatic adenocarcinoma, both Stim1 and Orai1 mediate SOCE and have a pro-survival anti-apoptotic role, as Orai1 and/or Stim1 silencing by siRNA enhance 5-Fluoro-Uracile (5-FU) and gemcitabine induced apoptosis [30]. Mechanisms by which the Orai/Stim complex affects chemoresistance include MDR transporters and intracellular signalling pathways (Figure 1). The overexpression of the transporter MDR1 is one of the best known mechanisms by which breast cancers cells develop chemoresistance [32]. It has notably been shown that high salts were able to induce MDR1 mediated treatment resistance in breast cancer cells through SOCE [33]. It has also been shown that Orai1 and Stim1 expression are increased in chemoresistant ovarian carcinoma cells, associated to an increase of SOCE. During the acquisition of the chemoresistant phenotype, Orai1 expression is upregulated by the Akt signalling pathway, leading to an increase of SOCE [34]. Furthermore, Orai1 expression is also elevated in hepatocellular carcinoma (HCC) tissues, and contributes to a chemoresistant phenotype. Orai1-mediated Ca^2+^ entry is able to block 5-FU-induced autophagic cell death in HepG2 cells via activation of the PI3K/Akt/mTOR signalling pathway [35]. In human gastrointestinal stromal tumors, Orai1 is able to mediate tumor-promoting SOCE via c-KIT and the ERK pathway [36]. Whilst far less studied than its sibling Orai1, Orai3 protein deserves special attention, because of (i) its exclusive presence in mammals [37], (ii) its receptivity to pharmacological modulation [38], and (iii) its recent emergence in the cancer field, especially in breast cancer. Many data establish now a clear link between Orai3 and chemoresistance (Figure 1). Orai3 was primarily described as involved in proliferation, cell cycle progression, and Estrogen Receptor positive (ER^+^) breast cancer cells survival [39]. In these cells, SOCE is dependent on Orai3 expression, whereas Estrogen Receptor negative (ER^−^) cells depends on Orai1 [40]. In such ER^−^ cells, Bhattacharya et al. [41] have shown that Orai3 and Stim1 were able to mediate TGF-β induced expression of Snai1, a transcriptional repressor protein known to mediate Epithelial to Mesenchymal Transition (EMT), a cell state transition process implicated in drug resistance [32]. This process was AKT and NF-κB (p65)-dependent [41]. In ER^+^ breast cancer cells, Orai3-dependent survival was found to be transduced—at least in part—via the ERK and c-myc pathway [42]. Overexpression of Orai3 protein in ER^+^ breast cancer cells revealed consistent chemoresistance against cisplatin, 5-FU and paclitaxel treatments. The main effect of Orai3 upregulation was a SOCE-induced downregulation of the tumor suppressor protein p53, leading to reduced apoptosis [43]. The complete pathway leading to p53 downregulation was extensively deciphered: increased SOCE is able to activate PI3K, but not Akt. Instead, the Sgk1 pathway is specifically activated, leading to Mdm2 activation and p53 downregulation. Sgk1 activation is also responsible for the inactivation of the Sek-1 kinase, and therefore to an hypo-phosphorylated and thus activated Nedd4.2 ubiquitin ligase, able to decrease p53 protein expression [43]. All these results are summarized in Figure 1 and in Table S1.

##### Reduced Resistance Conferred by the Overexpression of Stim and Orai Proteins

Resistance to apoptosis can also be acquired through a remodeling of the Ca^2+^ protein network that will reduce the likelihood of cytosolic Ca^2+^ overload. In androgen-independent, apoptosis-resistant prostate cancer cells, such remodeling was shown to involve reduced SOCE via downregulation of Orai1 or Stim1 proteins, and adaptation of the ER to the conditions of reduced Ca^2+^ storage and uptake [18,44]. In prostate cancer cells, low Orai1 expression was shown to contribute to the establishment of an apoptosis-resistant phenotype, irrespective of the apoptosis-inducing stimulus [17]. Orai3 overexpression in prostate cancer cells is able to impair the Orai1-mediated SOCE and causes prostate cancer cell resistance [45].

#### 2.1.2. Transient Receptor Potential (TRP) Channels

Transient Receptor Potential (TRP) channels are non-selective cation channels that can allow Ca^2+^ influx. The major groups of TRP channels include, among others, TRPM (Melastatin), TRPV (Vanilloid), and TRPC (Canonical). These channels have been associated with chemoresistance in many cancers since a decade [46,47].

TRPV—It has recently been shown that 5-FU induced breast cancer cell death was up-regulated by TRPV1 activation [48]: Ca^2+^ entry through TRPV1 is able to decrease chemoresistance. Nabissi et al. have shown that treatment with the TRPV2 agonist cannabidiol, by enhancing TRPV2 expression and activation, increased the chemosensitivity of human glioblastoma cells to the cytotoxic effects of various chemotherapeutic agents commonly used in the treatment of glioblastoma (temozolomide, carmustine and doxorubicin). Abrogation of chemoresistance was obtained by increased calcium influx through TRPV2 channel leading to a calcium-dependant cell death [49]. Moreover, cannabidiol and the proteasome inhibitor bortezomib synergise to increase cytotoxicity in multiple myeloma cell lines through TRPV2 activation [50]. Taken together, these data suggest that TRPV2 activation can potentiate the effects of cytotoxic drugs in various cancer types.

TRPM—In gastric cancer, Almasi et al. have shown that TRPM2 channel expression is negatively correlated with overall patient survival. Moreover, TRPM2 downregulation sensitized gastric cancer cells to paclitaxel and doxorubicin. Indeed, TRPM2, by activating both Akt and JNK signalling pathways, promote gastric cancer cell survival [51]. It has been shown that TRPM2 knockdown activates alternative pathway of cell death in breast cancer cells, leading to enhanced cytotoxicity after treatment with doxorubicin or *N*-methyl-*N*′-nitro-*N*-nitrosoguanidine [52]. In a similar manner, knockdown of TRPM8 was able to enhance epirubicin-induced apoptosis in osteosarcoma cells, and this effect was mediated through impaired regulation of intracellular Ca^2+^ concentration and the Akt-GSK-3β pathway [53].

TRPC—Downregulation of TRPC1 was shown to contribute to drug resistance in ovarian cancer, possibly by regulating autophagy [54]. Autophagy can be also mediated by TRPC5 and promotes drug resistance via CaMKKβ/AMPKα/mTOR pathway in breast cancer cells [55]. The TRPC5 channel has been largely associated with drug resistance in several cancers. TRPC5 can regulate Ca^2+^ homeostasis by forming a store-operated channel or a store independent channel. In addition, TRPC5-mediated Ca^2+^ entry is elicited by several physiological messengers, including reduced thioredoxin, protons, sphingosine-1-phosphate, lysophospholipids, NO and Ca^2+^ itself (see [56] for review). TRPC5 can confer chemoresistance to anticancer drugs in breast cancer. It has been shown that TRPC5 was essential for MDR1 induction in drug-resistant cancer cells [57], notably through activation of the transcription factor NFATc3 [58]. TRPC5 might even act as a non-invasive chemoresistance marker, since increasing circulating exosomes-carrying TRPC5 predicts chemoresistance in metastatic breast cancer patients [59]. In colorectal cancer, TRPC5 overexpression induces the Wnt/β-catenin signalling pathway and the up-regulation of MDR1 [60]. The same team demonstrated the essential role of glycolysis in TRPC5 induced chemoresistance in human colorectal cancer cells by maintaining Ca^2+^ homeostasis [61].

In HCC cells, inhibiting TRPC6 enhanced the efficacy of doxorubicin. Moreover, Ca^2+^ was found to be essential in mediating mechanisms of EMT, HIF-1α signalling and DNA damage repair, conferring multidrug resistance in HCC cells. Multidrug resistance was attributed to the sustained accumulation of intracellular free Ca^2+^ induced by TRPC6 overexpression. The pathway by which TRPC6 is able to induce multidrug resistance in these cells was demonstrated to be STAT3-dependent [62]. In a variety of other cancer cell lines (breast, prostate, bone, skin, lung), TRPC6 expression was on the contrary shown to lead to Ca^2+^-dependent apoptotic death and activation of apoptotic genes. Using the antineoplastic drug GaQ3 (an organic derivative of gallium), Madan et al. demonstrated that these apoptotic pathways were activated by the transcriptional upregulation of TRPC6 (and hence intracellular calcium) by p53 [63].

Mechanisms concerning the involvement of TRP channel in chemoresistance are provided in Table S1.

#### 2.1.3. Voltage-Gated Calcium Channels and Chemotherapy

Voltage-gated calcium channels (VGCC) are expressed throughout the body and perform several key physiological functions. In small cell lung cancer (SCLC), cells expressing the voltage-dependent calcium channel α2δ1 subunit (isolated from SCLC cell lines or patient-derived xenograft models) exhibit cancer stem cells characteristics and chemotherapeutic resistance. Treatment with etoposide enhanced the cell populations expressing α2δ1 in vitro and in vivo. Chemoresistance in these SCLC cell lines was shown to be mediated by the ERK pathway [64]. Moreover, the suppression of expression or activity of T-type Ca^2+^ channel induce apoptosis and hence allow resistance of ovarian cancer cells to chemotherapeutic treatments [65]. Apoptosis was accompanied by decreased Akt phosphorylation and alterations in FOXO and FOXM1 expression. Table S1 succinctly summarizes this information.

### 2.2. Intracellular Calcium Stores

Like extracellular calcium, intracellular calcium plays a decisive role in chemoresistance processes. Several intracellular organelles are implicated in such mechanisms, notably endoplasmic reticulum (ER) and mitochondria. Due to its closed proximity to the mitochondria, Ca^2+^ transport mechanisms from the ER to the mitochondria are allowed by the interaction of inositol 1,4,5-trisphosphate receptor (IP_3_R), the Glucose-Regulated Protein 75 and the Voltage-Dependent Anion Channel (VDAC) and the Mitochondrial Calcium Uniport (MCU). Since the MCU is the primary mediator of the mitochondrial Ca^2+^ uptake, its expression and function in cancer processes have been extensively investigated over the last ten years. Overexpression of MCU has been correlated to breast cancer progression [66], but its role in chemoresistance processes remains poorly understood. The amount of Ca^2+^ transferred from the ER to mitochondria impacts the sensitivity of cells to apoptotic stimuli and their resistance to apoptosis [67]. Moreover, it has been demonstrated that cancer cells escape cell death by lowering the ER Ca^2+^-store, due to a Ca^2+^ transfer between ER and mitochondria [68].

#### 2.2.1. Intracellular Ca^2+^ Channels Expression Is Associated to Chemoresistance

Due to the close interaction with mitochondria, and its role in the regulation of mitochondrial Ca^2+^ load, IP_3_R expression and/or activation have been associated to cancer cell survival and chemoresistance [69]. In bladder cancer cells, IP_3_R1 expression level has been inversely correlated to cisplatin-resistance: resistant cells express reduced IP_3_R1 level, and IP_3_R1-knockdown prevents apoptosis, leading to cisplatin resistance. On the other hand, IP_3_R1 overexpression in resistant cells induces apoptosis and increases sensitivity to cisplatin [70]. Conversely, high IP_3_R expression levels were associated to low resistance of epithelial pulmonary lung cancer cells to cisplatin treatment [71].

As a key regulator of the ER-mitochondrial interaction, the anti-apoptotic Bcl-2 protein is the subject of utmost attention. The Bcl-2-family members, which are generally divided into three categories (anti-apoptotic proteins, pro-apoptotic executioners and pro-apoptotic BH3-only proteins), act at two different intracellular compartments: the mitochondria and the ER [72]. Mechanistically, anti-apoptotic family members prevent cell death by sequestering the BH3 domains and by preventing their interaction with pro-apoptotic Bax/Bak proteins [73,74]. Small molecules (like BH3 mimetics) can disrupt this interaction, resulting in apoptotic cell death of cancer cells. Bcl-2 expression has been correlated to tumor growth enhancement and to chemoresistance [75] by increasing the passive Ca^2+^ leak from the ER [76]. Interestingly, the anti-apoptotic protein Bcl-2 expression level seems to be a determinant for cancer cell sensitivity to cisplatin [77]. In non-small cell lung cancer and bladder cancer, cisplatin sensitivity could be enhanced by downregulating Bcl-2 [78,79]. In addition, Bcl-2 downregulation in SK-OV-3 ovarian cancer cells increases Ca^2+^ levels in the cytosol and in the mitochondria, as well as the number of ER–mitochondrial contact points after cisplatin treatment, thereby increasing the sensitivity to the chemotherapeutic agent [80].

#### 2.2.2. Chemotherapy Modulates Intracellular Ca^2+^ Channels Activity

Numerous studies have demonstrated that intracellular channels/transporters/pumps expression is altered in cancers, leading to intracellular Ca^2+^ homeostasis perturbation. However, the impact of chemotherapeutic agents on the activity of these intracellular Ca^2+^ channels is less described. Zhang et al. established that paclitaxel accelerates spontaneous Ca^2+^ oscillations by increasing the IP_3_R opening frequency in the presence of Neuronal Calcium Sensor-1, a Ca^2+^ binding protein [81]. Moreover, Boutin et al. have demonstrated that PKA-induced IP_3_R1 over-activation triggers [Ca^2+^]_ER_ decrease: this mechanism allows the prostate cancer cell line LNCAP to survive androgen deprivation [82]. Recently, Kang et al. demonstrated that Trifluoperazine, a well-known antipsychotic drug with anticancer effects [83,84,85,86], inhibits glioblastoma invasion by binding to the Ca^2+^-binding protein, calmodulin subtype 2 (CaM2). This binding dissociates CaM2 from IP_3_R1 and IP_3_R2 leading to massive and irreversible Ca^2+^ release from intracellular stores by IP_3_R1 and IP_3_R2 subsequent opening [87].

#### 2.2.3. Signalling Pathways Involved in Chemoresistance Related to Intracellular Ca^2+^ Channels

**Figure 2:**
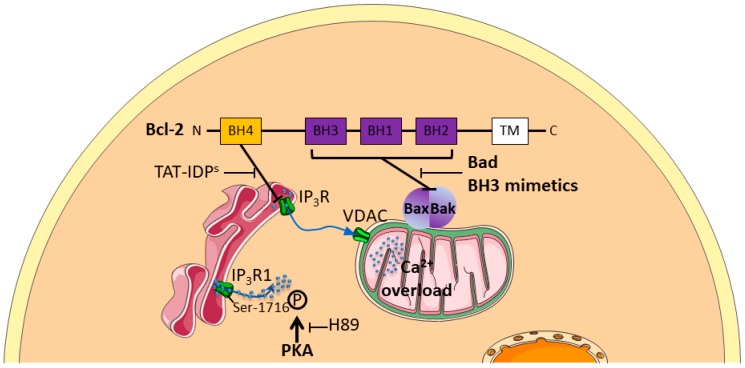
Schematic representation of intracellular Ca^2+^ pathways involved in chemoresistance. Anti-apoptotic Bcl-2 protein enhances chemoresistance by a dual role at both the Endoplasmic Reticulum (ER), by inhibiting Ca^2+^ depletion through Inositol 1,4,5-trisphosphate Receptors (IP_3_Rs), and the mitochondria by inhibiting apoptotic complex Bax/Bak formation. TAT-fused inositol 1,4,5-trisphosphate receptor-derived peptide (TAT-IDP^S^), by inhibiting BH4 domain, enhances Ca^2+^ efflux from the ER through IP_3_Rs to mitochondrial Voltage-Dependent Anion Channel (VDAC).

Mechanisms of intracellular Ca^2+^ pathways involved in chemoresistance are poorly understood, but few teams have succeeded in deciphering the communication between ER and mitochondria in such processes. In ovarian cancer cells, TAT-fused inositol 1,4,5-trisphosphate receptor-derived peptide (TAT-IDP^S^), which targets the BH4 domain of Bcl-2, enhances the cytotoxicity of cisplatin by stimulating Ca^2+^ efflux from the endoplasmic reticulum (ER) into the cytosol and the mitochondria, which further increased cisplatin-induced ER stress-mediated apoptosis by enhancing calpain-1 expression and by activating the mitochondrial apoptotic pathway [88]. Conversely, Bcl-2 attenuates cisplatin-induced Ca^2+^ release from the ER into the cytosol and the mitochondria, thus inhibiting cisplatin-induced ER stress-mediated apoptosis and activation of the mitochondrial apoptosis pathway. In this context, decreased ER mitochondrial crosstalk is responsible for Bcl-2 attenuation of cisplatin-induced mitochondrial Ca^2+^ accumulation in SKOV3 cells [89]. All these results are summarized in Figure 2 and compiled in Table S1.

Chemoresistance mechanisms have thus been described through phosphorylations inducing overactivation of IP_3_R1. Various pharmacological strategies by blocking Bcl-2 inhibition (TAT-IDPs or Bad/BH3 mimetics) or PKA inhibitors (such as H89) have already been suggested to counteract such chemoresistance processes [88,89].

#### 2.2.4. Intracellular Ca^2+^ Channels: Targets to Overcome Chemoresistance

Because of their inaccessibility to pharmacological agents, intracellular channels or receptors do not appear as priority target to overcome chemoresistance. Nevertheless, it has been shown that BH3 binding and the amount of Bim scaffolded by anti-apoptotic Bcl-2 proteins can be used as a predictive marker for the apoptotic response of cancer cells to chemotherapy [90]. It is proposed that cancer cells, in which the mitochondria contain high levels of Bcl-2 and Bim, are most sensitive to toxic stimuli, including chemotherapeutic drugs [91,92]. Hence, from these studies, it seems that the mitochondrial apoptotic priming can predict the tumor response to cytotoxic chemotherapy [91,92]. The mitochondrial priming state is thereby not the same in all malignancies [93]. For instance, cancers that are highly primed are those that respond most favorably to chemotherapy (e.g., blood cancers, including Chronic Lymphocytic Leukemia), whereas those that are unprimed respond poorly to chemotherapy (endometrial and renal cell carcinomas, serous borderline tumors) [90]. In the latter cases, the therapeutic window for using chemotherapy is very limited [72]. The number of studies published on the role of intracellular calcium channels or receptors in tumoral progression has increased over the last ten years, and may lead to them being considered as new targets for chemotherapy.

## 3. Potassium Channels

Among ion channels, potassium channels compose the largest family, presenting 78 sequences coding for alpha pore forming subunit [94]. Besides regulating many physiological functions (membrane potential, cell volume, excitability…), their involvement in cancer progression is now clearly established (e.g., proliferation, migration or invasiveness capacities, apoptosis resistance and angiogenesis, see [95,96,97] for review). By using a K^+^ ionophore, amphotericin B, the study reported by Sharp et al., was one of the first showing the importance of K^+^ flux in cisplatin resistance in ovary carcinoma [98]. In the 2000s, Liang et al. showed that cisplatin induced biophysical modifications of the membrane (lipid composition, fluidity), and cisplatin-resistant epidermal carcinoma cells present hyperpolarizing membrane potential compared to the sensitive parental cells [99]. Moreover, Marklund et al. [100,101] reported that both reduction of intracellular K^+^ concentration or addition of K^+^ flux modulators (amphotericin B and bumetanide) regulated cisplatin-induced apoptosis. These early results suggested that the activity of K^+^ channel was associated to therapeutic resistance. Since this demonstration, numerous groups detailed more precise mechanisms involving K^+^ channel in chemoresistance.

### 3.1. Correlation between the Expression Levels of K^+^ Channels and Sensitivity to Chemotherapeutic Drugs

Several studies have reported a correlation between the expression levels of K^+^ channels and the sensitivity to cytotoxic drugs. Both overexpression and reduction of K^+^ channels expression are involved in chemosensitivity. For example, using big data analysis, Liu et al. demonstrated an association between the reduction of *KCNN3* expression (a gene encoding for the SK3 K^+^ channel) and drug resistance of ovarian cancer [102]. Similar associations were reported on the classically used cisplatin [103]. Indeed, the decreased KCNMA1 (also referred to as BKCa—Calcium activated large conductance potassium channel—or maxi K channel) expression (involving potentially miR-31) was described to be associated to less sensitivity to this platinum-based chemotherapy in ovarian cell lines. In the same way, an association between the reduction of hERG (Kv11.1) expression and increased resistance to vincristine, paclitaxel and hydroxy-camptothecin is reported for different cancer cells [104]. Using similar approaches, Han et al. demonstrated that the voltage activated potassium channel Kv1.5 could participate in the cellular response of gastric cancer cells to adriamycin, and that a downregulation of the channel expression could promote the multidrug phenotype of these cells [105]. Additionally, the Leanza group demonstrated, in several cancer cell lines, a similar relationship between reduced expression of Kv1.1 and Kv1.3 and decreased sensitivity to drugs, provoking mitochondrial-induced apoptotic death [106]. Another study conducted using small cell lung carcinoma cells demonstrated a relation between K^+^ channel expression profile and drug resistance [107]. More precisely, authors demonstrated that the expression level of BKCa and Kv channels was inversely correlated to the MRP1 expression levels. They hypothesized that this observation could be a consequence of exposition to doxorubicin, which modulate the transcription factor c-jun (known to affect expression of both MRP1 and Kv channels). In this work, the relationship between K^+^ channel repression and chemosensitivity should be an acquired mechanism of resistance.

On the contrary, there are different cases described with association between overexpression and resistance to therapy. For example, an association between an upregulation of hEag1 (Kv10.1) and TWIK-2 channels and an increase of cisplatin resistance was shown by Liang et al. [108]. However, these authors described that channels are overexpressed but they did not obtain mechanistic association between the channel function and the efficiency of cisplatin in the epidermal and liver carcinoma cells tested. Arcangeli’s group showed higher levels of KCa3.1 and Kv11.1 channels in cisplatin-resistant colorectal cancer cells compared with their cisplatin-sensitive counterparts. In resistant cells, the treatment by riluzole (an activator of KCa3.1 and also an inhibitor of hERG) overcomes cisplatin resistance in both in vitro and in vivo models [109]. Pardo’s group demonstrated also that, depending on the clinical status, acute myeloid leukemia cells could express hEag1 and the inhibition of this channel improves the apoptosis induction by different chemotherapeutic drugs, suggesting the involvement of this channel in basal resistance [110]. hEag1 was also involved in chemoresistance in ovarian cancer cells. By using immunohistochemistry on tissue samples from patients treated with cisplatin-based adjuvant chemotherapy, Hui et al. found that a decreased hEag1 expression was correlated with a favorable prognosis and also predicts higher sensitivity to cisplatin treatment [111]. They also found that hEag1 silencing facilitated the sensitivity of ovarian cancer cells to apoptosis induced by cisplatin through the NF-κB pathway. They thus proposed hEag1 as a potential indicator to predict chemosensitivity.

### 3.2. Chemotherapy Modulates K^+^ Channel Activity

Chemotherapy can also alter the activity of K^+^ channels without affecting their expression. Chemotherapy drugs may increase or decrease the K^+^ channel activity depending on cancer types and drugs nature. In human lung epidermoid cancer cells, cisplatin activates the KCa3.1 channel without affecting its expression levels [112]. This activating effect of cisplatin on KCa3.1 may be due to the increase in intracellular calcium concentration by cisplatin. Results from our laboratory demonstrated similar implication of intracellular calcium concentration on chemoresistance in breast cancer ([43], section Ca^2+^ channel). In addition, it has also been observed in human carcinoma HeLa-S3 cells, that Ca^2+^ influx through Ca^2+^ channels is necessary for cisplatin-induced activation of a Ca^2+^-dependent K^+^ channel inhibited by charybdotoxin (BKCa channel) [113]. Also, Jirsch et al. presented in small cell lung cancers cells an increase activity of inwardly rectifying potassium channel associated to increased resistance [114]. The reduction of the activity of KCa3.1 has been demonstrated in both glioma cells (13-06-MG) and colon cancer cells (LoVo) by an oxaliplatin treatment, but not with cisplatin. Indeed, oxaliplatin decreases the KCa3.1 channel open probability [115].

### 3.3. Signalling Pathways Involved in Chemoresistance Related to K^+^ Channels

Some groups tried to further detail the cellular mechanisms linking K^+^ channels to chemotherapeutic resistance. Chemotherapeutic resistance could be assigned to numerous cellular processes [116]. Among them, links with K^+^ channels were reported in the modification of apoptosis regulation, feedback regulation through miRNA or even the relationship between tumour and microenvironment cells.

**Figure 3:**
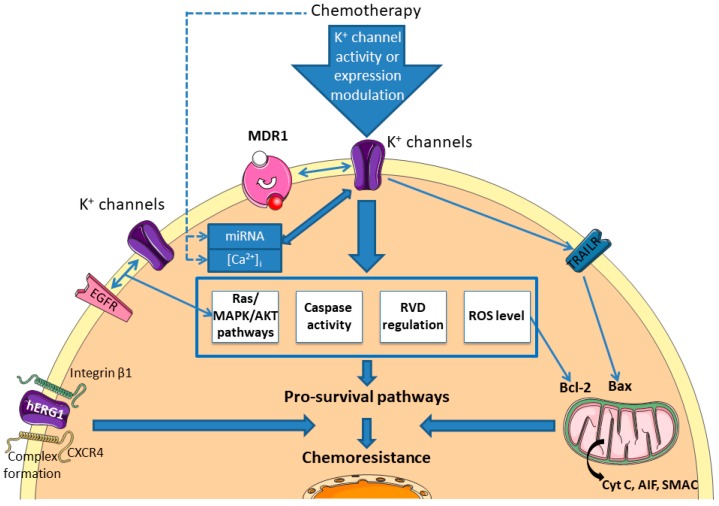
Chemoresistance pathways involving K^+^ channels. K^+^ channels could modulate the sensitivity to different chemotherapies by regulating pro-survival pathways (intrinsec or extrinsec apoptosis pathways, volume regulation, Akt/RAS/MAPK pathways, interacting with receptors or transporters). MDR: Multidrug Resistance Protein; EGFR: Epidermal Growth Factor Receptor; CXCR4: C-X-C Chemokine Receptor type 4; TRAILR: TRAIL Receptor; Cyt C: Cytochrome C; AIF: Apoptosis Inducing Factor; SMAC: Second Mitochondria-derived Activator of Caspases; RVD: Regulatory Volume Decrease.

It is now well known that K^+^ channels are involved in the regulation of the apoptosis mechanism through the control of mitochondrial membrane potential, the regulation of cell volume and the availability of K^+^ regulating caspase activity [117]. Indeed, different studies report that K^+^ channels are located in the mitochondrial membrane where they are involved in mitochondrial functions. Checchetto et al. reviewed the role of the major mitoK^+^ channel types described (Kv1.3, Kv1.5, K_ATP_, BKCa, KCa3.1, TASK3) and their involvement in cell death [118]. For example, the calcium-activated potassium channel KCa3.1 has been linked to the melanoma cell response in the presence of vemurafenib [119]. It was previously demonstrated that this BRAF inhibitor promotes apoptosis through a ROS-dependent pathway, which is prevented by the α-tocopherol. In the study by Bauer et al., it was demonstrated that the combination of TRAM-34 with vemurafenib induced an apoptotic pathway presenting general control through the anti-apoptotic and pro-apoptotic Bcl-2 family. Additionally, to the modification of MMP’s functions, they showed that early events are the production of ROS, which is crucial in the apoptotic cascade of this model. TRAIL (TNF-Related Apoptosis-Inducing Ligand) is a recent therapeutic opportunity to treat aggressive melanoma that presents high selectivity to cancer cells but also applicability limitations due to development of resistance. Quast et al. showed that the KCa3.1 inhibition by using TRAM-34 could also overcome the resistance to TRAIL in melanoma cell lines [120]. The same authors also deciphered the apoptotic pathway, which involves activation of caspases 3, 8 and 9, modification of the mitochondrial membrane potential, a dependency to Bax, and the release of mitochondrial factors like cytochrome c, AIF and SMAC (Figure 3). It is worth noting that the cellular response involving K^+^ channel is very specific of the cellular type involved. KCa3.1 channel can also be involved in cisplatin sensitivity. Using the epidermoid cancer cell model, Lee et al. demonstrated that the activity of KCa3.1 is necessary to the cisplatin-induced death [112]. In this model, authors showed that the channel participates in the control of the Regulatory Volume Decrease (RVD) and caspase3/7 activity, two processes implicated in the apoptotic mechanism. In the same manner, it has been reported that the activity of the hERG channel is important for cisplatin-induced death of gastric cells [121]. This work demonstrated that cisplatin exposure increases the expression of hERG and that the activity of this channel is necessary in cisplatin-induced death of the different cell lines used. In addition, authors demonstrated that the activity of the channel is important to the caspase dependent apoptotic pathway through the regulation of Bax/Bcl-2 axis and active caspase 3. Their results were confirmed by using animal model where they also demonstrated the involvement of the hERG channels in the cisplatin-induced apoptosis. Using cisplatin-resistant colorectal cancer cell, Pillozzi et al. showed that the combined activation of KCa3.1 and inhibition of Kv11.1 by riluzole reduced the level of p-Akt and p-ERK and increased caspase-3, demonstrating a synergic effect with cisplatin to overcome the resistance to this chemotherapeutic agent [109]. In addition, they demonstrate that the modification of the channel activity allowed an increase in the incorporation of platinum salt in the cell to promote its activity.

To the best of our knowledge, only one report described the implication of K^+^ channel in the modulation of the EGFR-Ras-Raf signalling pathway and an association with chemosensitivity (Figure 3). In side population of lung cancer stem cells, Choi et al. demonstrated that the use of different K^+^ channel modulators affect the gefitinib efficiency to kill lung cancer cell line [122]. Indeed, application of 4-aminopyridine (Kv blocker), TetraEthylAmmonium (Kv blocker) or flupirtine (Kv7 opener) in combination with gefitinib decreases drastically cell viability compared to the use of the compound alone. This effect is correlated to the reduction of p-EGFR, total Ras, p-Raf and p-ERK1/2, compared to the gefitinib condition alone.

Among the different molecular mechanisms described in the literature, some articles reported a close link between miRNA, K^+^ channel, and chemoresistance (Figure 3). For instance, by using immunohistochemistry, different SCLC cell lines resistant to chemotherapeutic drugs and preclinical in vivo model, Liu et al. demonstrated the involvement of the Kir2.1 channel in drug resistance [123]. First, they described that overexpression of the channel is responsible for the increase of the MRP1/ABCC1 protein in the different cell lines tested and that the level of the channel and of the MDR are correlated with clinical parameters. Second, they evaluated different putative regulators of the channel. They have shown that (i) Kir2.1 expression is modulated by the RAS/MAPK pathway, (ii) Kir2.1 channel is directly regulated by the miR-7, already described in the chemoresistance process. They thus described that miR-7 (downregulated in SCLC) and RAS/MAPK pathway promote directly the expression of Kir2.1, which that is a crucial actor in the resistance to cisplatin, adriamycin and etoposide in the different models used in this study. Similarly, it was described in glioblastoma cell line that reduced expression of miR-296-3p is concurrent to the increase expression of hEag1 and these two modifications are associated to an increased resistance to temozolomide, etoposide, and imatinib [124]. The authors demonstrated here that overexpression of the miRNA drastically decreases the level of hEag1 and restores sensitivity to the drugs. On the contrary, the use of antago-miR to mimic the cancerous conditions promotes resistance in association with an increase of the hEag1 transcription.

A study conducted by Pillozzi et al. reported an interesting relationship between cancer cells and the microenvironment, more precisely acute lymphoblastic leukemia (ALL) cells and bone marrow mesenchymal cells (MSC), involving hERG1 channel and resistance to chemotherapeutic drugs [125]. They demonstrated that the complex involving β1-integrin subunit, CXCR4 and hERG1 channel is important for interactions between MSC and ALL cells, and regulates protection against drugs of ALL cells by MSC. They probed deeper into the mechanism by using different hERG1 inhibitors and demonstrated that this channel is crucial for the protection of ALL by microenvironment cells. These results are summarized in Figure 3 and in Table S1.

### 3.4. K^+^ Channels: Targets to Overcome Chemoresistance

Evidence reported previously suggest that K^+^ channels could be interesting adjuvant targets to fight against the resistance of cancer cells. For instance, astemizole, an inhibitor of Kv10.1, has been evaluated in combination with gefitinib on lung cancer cells [126]. In their models, the authors demonstrated that this association enhances the mortality of cancer cells compared to the application of separate molecules. To pursue these demonstrations, different research teams evaluate this type of combination on preclinical models based on in vivo analyses. For example, the concomitant activation of KCa3.1 and inhibition of hERG1 allow to overcome cisplatin resistance in colorectal cells [109].

Interestingly, some inhibitors of K^+^ channels can induce cell death, bypassing the classical mechanisms of chemotherapies. Indeed, Leanza et al. demonstrated that the use of mitoKv1.3 permeant inhibitors could affect this channel at the mitochondrial level, provoking the release of cytochrome C and eliciting the death of cancer cells [127]. This approach could be very interesting to bypass the barrier of the deregulation of the Bax/Bcl-2 pathway.

All elements presented through this state of research about K^+^ channel implicated in chemoresistance suggest that it should be interesting to clinically evaluate specific modulators of this class of ion transporters.

## 4. Magnesium Channels

**Figure 4:**
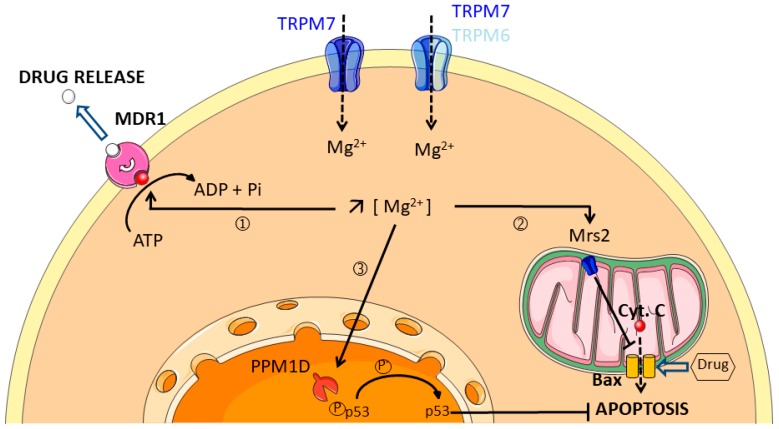
Multidrug resistance is associated with an increase of intracellular free Mg^2+^. Intracellular free Mg^2+^ could promote multidrug resistance through three main potential mechanisms: ① Mg^2+^ Џbinds to MDR1 leading to drug extrusion; ② Mg^2+^ enters into the mitochondria through Mrs2 channel leading to Bax inhibition and resistance to apoptosis, ③ Mg^2+^ activates the nuclear PPM1D phosphatase which alters p53 stabilization and protects cancer cells against apoptosis. Chemoresistant cancer cells have a lower Mg^2+^ influx and lower amounts of TRPM6 and TRPM7 channels at the plasma membrane.

Magnesium (Mg^2+^) is the second most abundant cation in the cell. The main role of Mg^2+^ is to act as a coenzyme in virtually all the biochemical catalytic reactions [128]. Although Mg^2+^ status is not systematically assessed in clinical examination, recent epidemiological data suggest that Mg^2+^ deficiency can be linked to an increase in cancer risk. For example, 100 mg daily decrease of Mg^2+^ intake is associated with a 24% increase in the incidence of pancreatic cancer [129]. Moreover, increase of Mg^2+^ intake is also associated with a reduction of colorectal adenoma risk [130]. Mg^2+^ homeostasis of the cell is mainly regulated by the expression of Mg^2+^ transporters at the plasma membrane. A growing number of studies show that Mg^2+^ transporters could be involved in cancer progression. Importantly, Mg^2+^ cytosolic concentration is increased in doxorubicin-resistant colon cancer cells while the Mg^2+^ influx is reduced when compared to sensitive cancer cells [131]. This lower Mg^2+^ influx is due to the downregulation of TRPM6 and TRPM7 Mg^2+^ channel expression. Interestingly, TRPM7 silencing in drug sensitive cells shifts their phenotype toward a more resistant one. These data suggest that drug resistance is associated with alteration of Mg^2+^ homeostasis through TRPM7 regulation in colon cancer cells (Figure 4). However, the role of TRPM7 in cancer cell resistance has never been studied, even if its expression is clearly linked to cancer progression and reduced survival in breast and pancreatic cancer patients [132,133]. It has also been shown that human mitochondrial Mrs2 protein expression is upregulated in a multidrug-resistant gastric cancer cell line compared to its non-resistant counterpart [134]. Mrs2 is located in the mitochondria inner membrane and is responsible for Mg^2+^ transport in the mitochondria of mammals. Interestingly, Mrs2 expression seems positively correlated with the multidrug resistance of gastric cancer cells in vitro and in vivo. In gastric cancer cells, Mrs2 overexpression inhibits adriamycin-induced apoptosis, probably by suppressing Bax-induced cytochrome-C release by mitochondria. Moreover, p27 is downregulated while cyclin D1 is upregulated following Mrs2 overexpression, leading to gastric cancer cell proliferation enhancement. Although these data suggest that Mrs2 may be a promising target against multidrug resistant gastric cancer, the role of mitochondrial Mg^2+^ has not been yet elucidated (Figure 4).

The only described mechanism by which magnesium is able to confer resistance was published some 30 years ago: Mg^2+^ was reported as essential for vincristine binding to the plasma membrane of multidrug-resistant human myelogenous leukemia K562 cells [135]. The binding was observed at 100 µM Mg^2+^, and reached a maximum effect in the 5–10 mM Mg^2+^ range. The candidate for drug efflux from K62 cells is the ATPase MDR1. The ATPase activity of MDR1 is indeed dependent on Mg^2+^, but can also be modulated by other divalent cations, including manganese (Mn^2+^) and cobalt (Co^2+^). On the other hand, calcium (Ca^2+^), zinc (Zn^2+^), nickel (Ni^2+^), cadmium (Cd^2+^) and copper (Cu^2+^) inhibit the Mg^2+^-catalyzed ATP hydrolysis [136].

Calcium blockers verapamil and nicardipine are able to inhibit both vincristine binding to the plasma membrane of K52 cells and drug efflux in a competitive manner, independently of the surrounding calcium concentration [136,137]. Taken together, these findings confirm that unlike Ca^2+^, Mg^2+^ is essential for active transport of drug outside the cell [137]. Mg^2+^ is also required for the catalytic activity of the Protein Phosphatase Magnesium-dependent 1D (PPM1D) that confers cisplatin resistance in ovarian and cervical cancer cell cells by deactivating p53 [138]. PPM1D promotes drug resistance in gynecological malignancies by acting as a downstream target of Akt. Moreover, recent studies show that mutations of PPM1D in hematopoietic stem cells confer a selective advantage to resist apoptosis-induced by cytotoxic treatments in a context of clonal hematopoiesis prior to malignancy development [139,140]. On the other hand, clinical data demonstrate that patients treated with cetuximab and panitumumab, monoclonal antibodies targeting the epithelial growth factor receptor (EGFR), develop hypomagnesemia [141,142,143,144,145]. All these results are summarized in Figure 4 and in Table S1. These data suggest that hypomagnesemia is a side-effect of chemotherapy that is associated with better clinical benefits. However, total serum Mg^2+^ level is measured for hypomagnesemia assessment in clinical studies and does not necessarily represents the bioactive fraction of Mg^2+^ into cells and tissues.

It is tempting to speculate that any modification of the Mg^2+^ transporter expression could disturb cytosolic Mg^2+^ homeostasis leading to enhanced drug resistance in cancer cells. However, future investigations are needed to better understand how Mg^2+^ transporters are expressed and could eventually coordinate during cancer progression from primary tumor to metastatic and multidrug resistant cancer.

## 5. Chloride Channels

The role of selective anion channels, more specifically chloride channels, has been well-described in physiological contexts and their implications in the excitability (neurons, skeletal, cardiac and smooth muscle), the regulation of cell volume, trans-epithelial transport, internal and external acidification, cell cycle progression and apoptosis are the subject of numerous studies ([146] for review). However, anion transporters were initially less studied in the cancer context until the discovery of some structural similarities with the MDR drug transporters involved in chemoresistance. For a decade, the number of reports has grown exponentially and different groups presented reviews about specific class of chloride channels (e.g., CLIC1 [147]; TMEM16A [148]). Although there is no official classification, the chloride channels are subdivided into five classes: ClC family (with 9 members), CFTR, Ca^2+^-activated chloride channels, Maxi-chloride channels and Volume Regulated Chloride (VRAC) channels [149]. Like other ion channels, chloride channels and subsequent signalling pathways are involved in tumor progression and aggressiveness through the regulation of cell motility, cell cycle progression or apoptosis resistance. Additionally, some groups revealed the implication of chloride channels in tumor resistance to chemotherapy. Two major trends have recently emerged: on one hand, expression or activity reductions of VRAC induce resistance to the pro-apoptotic molecules by regulating the apoptotic volume decrease (AVD), and on the other hand, different chloride channels are upregulated and trigger pro-survival pathway or promote different mechanisms to reduce drugs activity.

### 5.1. Volume Regulated Chloride Channel Reduction and Promotion of Chemoresistance

**Figure 5:**
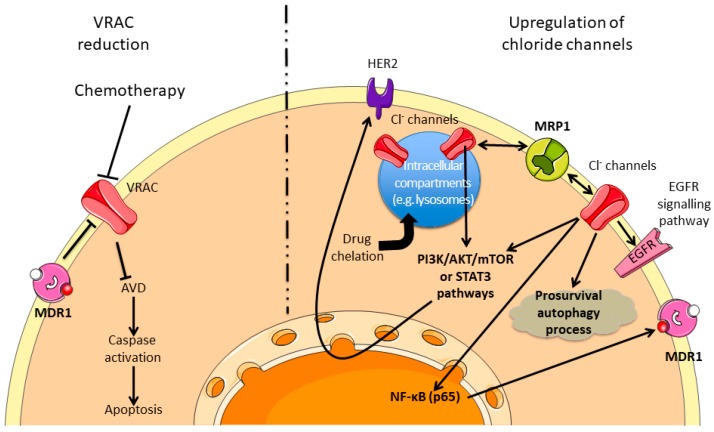
Chemoresistance pathways involving Cl^−^ channels. Cl^−^ channels can modulate the sensitivity to different chemotherapies by inhibiting the AVD or by modulating the prosurvival pathways and the drugs availabilities. The dashed line separates two virtual cells presenting either reduction activity of Cl^−^ channels (left part) or an upregulation of Cl^−^ channels (right part). MDR1: Multidrug Resistance 1; VRAC: Volume Regulated Chloride Channel; AVD: Apoptosis Volume Decrease; HER2: Human Epidermal growth factor Receptor 2; MRP1: Multidrug associated Resistance Protein 1; EGFR: Epidermal Growth Factor Receptor.

One of the first events at the onset of the apoptosis is a variation of the cell volume due to a modification of the ions (classically K^+^ and Cl^−^), solutes and water transports, which precedes caspases activation. Among the different transporters involved in this mechanism, the implication of VRAC has been well established. In addition, it is strictly demonstrated that different chemotherapeutic drugs induce cancer cell apoptosis. The reports of activity or expression reduction of the VRAC molecular support in relation with chemotherapy sensitivity are obvious (Figure 5, left part). For instance, it has been described that doxorubicin could affect VRAC current in MCF-7 cells and the cell response is based on the relationship between MDR1 expression and I_(Cl-swell)_ activity [150]. In addition, by using epidermoid cancer cell lines sensitive or resistant to cisplatin, Okada’s group demonstrates the involvement of VRAC in the response to this drug [151,152,153]. The association of this type of current to drug resistance has also been reported in lung, nasopharyngeal carcinoma or anthracyclin MDR cell line [154,155,156] and was the object of specific reviews to this subject [157,158]. Interestingly, the molecular identification of the VRAC channel, the LRCC8 family [159], occurred recently and new understanding appeared with this highlight. Indeed, it has been shown that the specific subunit composition of the VRAC current affects differentially the sensitivity to chemotherapeutic compounds [160].

### 5.2. Upregulation of Chloride Channels and Chemoresistance

As previously described, chemotherapy resistance is based on the modification of different cellular processes (e.g., apoptosis resistance, autophagy regulation, and MDR modulation). By affecting these processes, chloride channels have also been demonstrated to be major regulators of drug resistance (Figure 5, right part). It has been thus demonstrated that the overexpression of CLIC1 or ANO1 are correlated to the resistance of glioblastoma cancer stem cells, ovarian or breast cancer [161,162,163,164]. In another way, Bill et al. demonstrated that ANO1 channel could be functionally linked to the EGFR pathway and its expression level could be used as predictive biomarker to anti-EGFR therapy in head and neck squamous cancer [165].

Chloride channel can be located at the plasma or intracellular membrane level. Weylandt et al. demonstrated that ClC-3 could participate in the acidification of intracellular compartment in order to promote the chelation of the etoposide in neuroendocrine tumor cells and consequently reduce the activity of this compound [166]. Similarly, it was shown that ClC-3 could participate in cisplatin resistance in erythroleukemia or glioma models by modulating the pH of intracellular compartments, such as lysosome [167,168]. In addition, Fujito et al. demonstrated that ClC-3 and ANO1, by regulating intracellular chloride level, could control the transcription of HER2 in breast cancer cells through the PI3K/Akt/mTOR or STAT3 pathways, depending on the cell/channel type promoting the resistance to anti-HER2 therapies [169]. ClC-3 has also been demonstrated to participate in cisplatin resistance by regulating the Akt pathway and the autophagy process of glioma cells [170] or to promote the decreased answer to different compounds by regulating the overexpression of MDR1 by NF-κB and p65 signalling [171]. CLC-5 has been demonstrated to promote bortezomib resistance in multiple myeloma cell lines by increasing the pro-survival autophagy and inhibiting the Akt-mTOR pathway [172]. In choriocarcinoma models, sensitive or not to methotrexate and floxuridine, CLIC1 channel has been shown to be overexpressed in resistant cells and linked with the upregulation of MRP1 in order to promote drug resistance [173]. All these results are summarized in Figure 5 and Table S1.

## 6. Other Ion Channels

As previously described, the associations between Ca^2+^, K^+^, Mg^2+^ or Cl^−^ channels with chemoresistance present an increasing number of iterations in the bibliography. However, little information is available regarding the involvement of other channels, water, and solute transporters in the literature. It is however worth mentioning that voltage-gated sodium channels and ASIC channels have been associated with chemoresistance.

### 6.1. Voltage-Gated Sodium Channel in the Chemoresistance Process

This class of ion channel is known to participate in tumor progression by improving cell invasiveness capacity in different tumor types (e.g., breast, colorectal, prostate cancer [174,175,176]). However, Gerard et al. described that the NG108-15 cells (“neuroblastoma x glioma” hybrid cells), that are resistant to doxorubicin, show a 1.66-fold increase in Na^+^ conductance when compared to the parental cells [177]. These results are in good agreement with those obtained by Yamashita et al., who showed that leukemia cells expressing a MDR phenotype present a bigger voltage-gated Na^+^ current compared to the sensitive ones [178]. In this study, they confirmed the association between voltage-gated Na^+^ current and MDR phenotype by using revertant cell lines (cell lines that lost the acquired resistance to drug) that did not maintain the Na^+^ conductance.

### 6.2. ASIC Channels in the Chemoresistance Process

It has recently been demonstrated by Zhang et al. that the ASIC1a channel participates in chemotherapy resistance of HCC [179]. ASIC1a is shown to be more expressed in HCC tissues, and the authors have deciphered a pathway involving the channel in the chemoresistance. They notably show that ASIC1a is expressed at the plasma membrane level, where it allows a Ca^2+^ influx, which regulates the PI_3_K/Akt pathway, protecting the cells against apoptosis in an extracellular acidic environment.

## 7. Conclusions

Chemoresistance is a major hurdle in cancer treatment leading to recurrence of cancer. Resistance is a complex phenomenon involving multiple mechanisms, including activation of signalling pathways, apoptosis resistance improvement, and increasing extrusion of therapeutic compounds, among others mechanisms. Although many drugs/therapies are now available in oncology, resistance to treatments impedes complete therapeutic successes and is responsible for mid- and long-term recurrence of the disease. Ion fluxes have been reported to modulate the response of cancer cells to several chemotherapeutic drugs [7]. Additionally, apoptosis regulation by ion channels is also well described ([180,181] for review) and consequently, it is interesting to understand their implication in the resistance to therapeutic compounds.

Although many papers report an association between chemoresistance and ion channel expression and/or activity, relatively few studies provide the complete mechanism of resistance. It appears thus necessary to improve knowledge about the different mechanisms involving ion channels, to enable potential new therapeutic associations (including ion channel modulators) to ideally overcome resistance to chemotherapeutic compounds.

**Figure 6:**
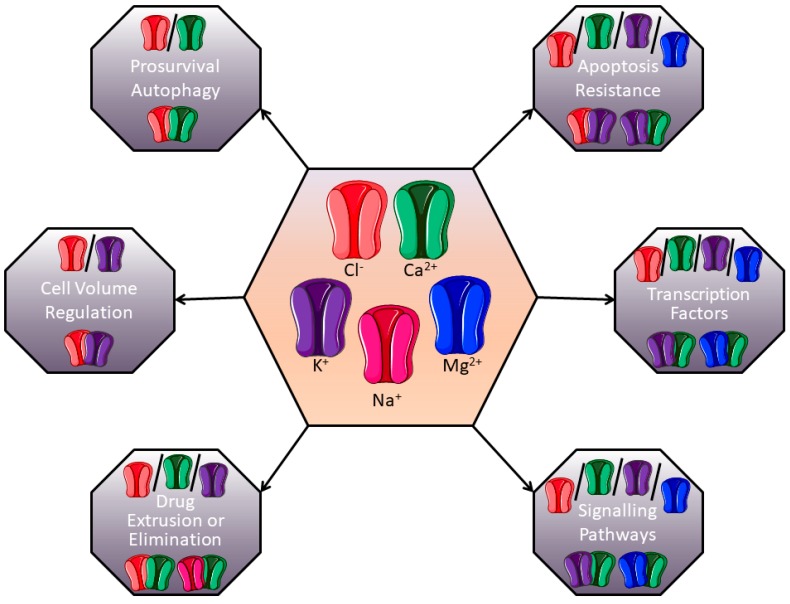
Putative channel associations involved in chemoresistance. Different ion channels could form complexes and modulate chemoresistance. In each grey box, the current description of channel associated-chemoresistance is presented in the light part, and examples of putative ion channels associations are suggested in the dark part.

Studies reported herein usually describe the implication of one channel in the regulation of chemoresistance processes. However, studies about complexes of ion channels are emerging to explain their involvement in the control of tumour progression. For example, our group demonstrated that the Kv10.1 potassium channel and the Orai1 calcium channel are associated to promote survival of breast cancer cells [182]. It was also demonstrated that the SK3 potassium channel, in association with TRPC1 and Orai1 calcium channels, regulated colon cancer cell migration [183]. Based on data from the literature, and since global ion channel expression is rather dynamically and interdependently regulated, we propose that ion channels-dependant chemoresistance processes are likely to depend on more complex mechanisms than only one channel and only one downstream signalling process (Figure 6). For example, K^+^ channels can be associated with Cl^−^ channels to regulate cell volume and modulate apoptosis resistance. In the same manner, the association of K^+^ channels with Ca^+^ ones could be very important in controlling intracellular signalling pathways or expression/repression of transcription factors. Future research in this field should thus take relationships between ion channels into account, and provide better description of the downstream molecular mechanisms involved in chemoresistance.

To conclude, this emerging field can highlight new targets or new evidence for overriding chemoresistance, but intensive work is still necessary to complete the list of actors involved, the interdependency between some of these actors, and their exact roles in chemoresistance processes by deciphering downstream signalling mechanisms more accurately. Ion channel blockers have been successfully developed over decades by pharmaceutical industries to treat cardiac or psychiatric disorders. Yet, their therapeutic potency has not been extensively investigated in clinical cancer therapy. Such ion channel blockers could, however, be very useful: for instance, verapamil, a Ca^2+^ channel blocker significantly increases the survival of patients with anthracycline-resistant metastatic breast carcinoma when given in association with chemotherapy [184]. Moreover, mibefradil dihydrochloride, a T-type Ca^2+^ channel blocker, acts as a radiosensitizer by potentiating the effect of hypofractionated radiation on patients with recurrent glioblastoma [185]. Others Na^+^ channel blockers, such as riluzole, have also been described in cancer therapy for patients with brain metastases originating from melanoma [186,187]. Hopefully, accumulating data on chemoresistance conferred by ion channels (most of which are summarized in the present review) will help repurpose ion channels modulators in clinical trials to improve cancer treatment.

## Supplementary Materials

The following are available online at https://www.mdpi.com/2072-6694/11/3/376/s1, Table S1. Ion channels and associated mechanisms/signalling pathways involved in chemoresistance.

## Abbreviations

AIF	Apoptosis-Inducing Factor
ALL	Acute Lymphoblastic Leukemia
ANO1	ANOctamin 1
ASIC	Acid-Sensing Ion Channel
AVD	Apoptosis Volume Decrease
Bax	Bcl-2-Associated X protein
Bcl-2	B-cell lymphoma 2
BKCa	Large conductance, Ca^2+^-activated K^+^ channels
CFTR	Cystic Fibrosis Transmembrane conductance Regulator
ClC	Chloride Channel
CLIC1	Chloride Intracellular Channel 1
CXCR4	C-X-C Chemokine Receptor 4
ER	Endoplasmic Reticulum
EMT	Epithelial to Mesenchymal Transition
HCC	HepatoCellular Carcinoma
hEag1	human Ether-à-Go-Go
hERG	human Ether-à-Go-Go-Related Gene
IP_3_R	Inositol 1,4,5-trisphosphate Receptor
MCU	Mitochondrial Calcium Uniport
MDR	MultiDrug Resistance
miRNA	micro-RNA
MMP	Matrix MetalloProteinase
MRP1	Multidrug Resistance-associated Protein 1
MSC	Bone Marrow Mesenchymal Cells
RVD	Regulatory Volume Decrease
SMAC	Second Mitochondria-derived Activator of Caspases
SK3	Small conductance, Ca^2+^ activated K^+^ channel 3
SOC channels	Store-Operated Calcium channels
SOCE	Store-Operated Calcium Entry
TAT-IDP^s^	TAT-fused inositol 1,4,5-trisphosphate receptor derived peptide
TRAIL	Tumor-necrosis-factor Related Apoptosis Inducing Ligand
VDAC	Voltage-Dependent Anion Channel
VRAC	Volume-Regulated Anion Channel
