# Supplementary material for: Ion Channels: New Actors Playing in Chemotherapeutic Resistance
Source: Cancers (Basel). 2019 Mar 16;11(3):376. doi: 10.3390/cancers11030376 (PMC6468599; doi:10.3390/cancers11030376)
Supplement: Supplementary file 1 [file cancers-11-00376-s001.pdf]

Supplementary materials: Ion Channels: New Actors Playing in Chemotherapeutic Resistance

Philippe Kischel, Alban Girault, Lise Rodat-Despoix, Mohamed Chamlali, Silviya Radoslavova, Hiba Abou Daya, Thibaut Lefebvre, Arthur Foulon, Pierre Rybarczyk, Frédéric Hague, Isabelle Dhennin-Duthille, Mathieu Gautier and Halima Ouadid-Ahidouch

Table S1: Ion channels and associated mechanisms/signalling pathways involved in chemoresistance.

Ion channels	Cancer models	Drugs	Mechanisms and signalling pathways	PMID
		**Calcium channels**		
STIM1	Osteosarcoma	Cisplatin	High activity (Increase of SOCE) associated with resistance to drug mediated apoptosis	28326487
Orai1	Pancreatic adenocarcinoma	5-FU<br>Gemcitabin	High activity (SOCE) due to an overexpression linked to an increase in both survival and resistance to apoptosis	24583265
	Ovarian carcinoma	Cisplatin	Overexpression associated with low sensitivity. Overexpression of Orai1 is regulated by the Akt signaling pathway	25015419
	Hepatocarcinoma	5-FU	Orai1 expression associated with chemoresistant phenotype<br>Orai1 mediated entry blocks 5-FU induced autophagy via the PI3K/Akt/mTOR signaling pathway	27878958
	Prostate cancer		Downregulation of Orai1 (or STIM1) lead to a decrease in SOCE and an adaptation of the ER in apoptosis-resistant cells	12086875<br>14685164
			Low expression of Orai1 contributes to the establishment of an apoptosis-resistant phenotype	21364678
Orai3	ER+ breast cancer	5-FU<br>Cisplatin<br>Paclitaxel	Orai3 overexpression revealed chemoresistance via an activation of PI3K pathway leading to a degradation of P53	29323264
	Prostate cancer		Orai3 overexpression impairs the Orai1-mediated SOCE and causes resistance	24954132
TRPV1	Breast cancer	5-FU	5-FU-induced cell death is up-regulated by TRPV1 activation	28795251
TRPV2	Glioblastoma	Temozolomide<br>Carmustine<br>Doxorubicin	Targeting of TRPV2 by an agonist increases the chemosensitivity of cells	23079154
	Multiple myeloma		TRPV2 activation increases cytotoxicity	24293211
TRPM2	Gastric cancer	Paclitaxel		29343514

		Doxorubicin	TRPM2 downregulation increases sensitivity to drugs. TRPM2 promotes survival by activating Akt/JNK signaling pathway	
	Breast cancer	Doxorubicin	Knockdown of TRPM2 enhances chemotherapy-induced cytotoxicity	26178079
		N-methyl-N'-nitro-N-nitrosoguanidine		
TRPM8	Osteosarcoma	Epirubicin	Knockdown of TRPM8 enhances chemotherapy-mediated apoptosis through the Akt/GSK-3beta signaling pathway	24391455
TRPC1	Ovarian cancer	Cisplatin	Downregulation of TRPC1 contributes to drug resistance by regulating autophagy	26647723
		Carboplatin		
TRPC5	Breast cancer	Doxorubicin	TRPC5-induced autophagy promotes chemoresistance to doxorubicin through CAMHHbeta/AMPKalpha/mTOR signaling pathway	28600513
			TRPC5 induces chemoresistance in breast cancer cells by upregulating MDR1 through activation of NFATc3	22988121
				24582564
	Colorectal cancer	5-FU	TRPC5 overexpression induces Wnt/beta-catenin signaling pathway and upregulates MDR1	25404731
			Glycolysis plays a role in TRPC5-induced chemoresistance	29463225
TRPC6	Hepatocellular carcinoma	Doxorubicin	TRPC6 overexpression lead to an accumulation of intracellular calcium which activates a STAT3-dependant signaling pathway and conferring multidrug resistance	27011063
		Cisplatin		
		5-FU		
	Multiple cancer models	GaQ3	TRPC6 expression lead to a Ca^{2+}-dependent apoptosis	23976973
VGCC alpha2delta1	Lung cancer	Etoposide	Expression associated to a chemotherapeutic resistance regulated by ERK signaling pathway	29437792
T-Type VGCC	Ovarian cancer	Cisplatin	T-Type calcium channel expression associated with chemoresistance through AK/FOXO/FOXM1 signaling pathway	26832797
IP3R1	Bladder cancer	Cisplatin	Reduced IP3R1 expression promotes chemoresistance to cisplatin	15608674
IP3R	Pulmonary cancer	Cisplatin	Elevated IP3R expression associated to low chemoresistance to cisplatin	19633366
		**Potassium channels**		
Kv1.1 and Kv1.3	Multiple cancer models	Staurospsorine, Ceramide and Cisplatin	Low expression associated to low sensitivity	23701546
Kv1.5	Gastric cancer	Adriamycin	Low expression associated to low sensitivity	17428690
hEAG1	Ovarian cancer	Cisplatin	High expression associated with high resistance	26079730

	Glioblastome	Telozomide, Etoposide and Imatinib	Reduced miR-296-3p expression increases hEAG1 to improve resistance	22999387
	Acute myeloid leukemia	Idarubicin, Etoposide, Cytarabine, Doxorubicin	High expression associated with high resistance	20105281
hERG/Kv11.1	Multiple cancer models	Vincristin, Paclitaxel, Hydroxy-camptothecin	Low expression associated to low sensitivity	15812674
	Gastric cancer	Cisplatin	Channel activity associated to caspase apoptotic pathway involved in cisplatin response	22020779
	Acute lymphoblastic leukemia		Regulation of chemoresistance through the association with bone marrow mesenchymal cells	21048156
hERG and TWIK-2	Epidermal and liver carcinomas	Cisplatin	High expression associated with high resistance	16309205
Ca2+-dependent K+ channel	Cervical cancer	Cisplatin	Channel activity associated to cisplatin response	17592515
SK3	Ovarian cancer		Low expression associated to low sensitivity	29901154
	Colorectal cancer	Cetuximab	K^+ channel inhibition potentiates Cetuximab efficiency *via* EGFR-AKT pathway modulation	27102434
KCa3.1	Lung epidermoid cancer	Cisplatin	Channel activity associated to cisplatin response through apoptotic pathway (RVD, caspase modulation)	18367588
	Glioma and colon cancer	Oxaliplatin	Channel activity associated to oxaliplatin response	26488725
	Melanoma	Vemurafenib	Channel inhibition potentiates apoptosis through regulation of Bcl-2 family	28151482
	Melanoma	TRAIL	Channel activity link to mitochondrial apoptosis pathway; KCa3.1 inhibition potentiates TRAIL efficiency	22723988
KCa3.1 and Kv11.1	Colorectal cancer	Cisplatin	High expression associated with high resistance; modulation of the AKT/ERK and the caspase 3 pathways	29161243
BKCa	Ovarian cancer	Cisplatin	Low expression associated to low sensitivity, association between miR-31 expression, BKCa expression and chemoresistance	26386726

BKCa (and Kv type channel)	SCLC[1]		Low expression associated to low sensitivity	16883578
Inwardly rectifying K+ channel	SCLC[1]		Increased channel activity associated to increased resistance	8395978
Kir2.1	SCLC[1]		Kir2.1 expression/activity increases MRP1/ABCC1 expression; Kir2.1 regulation by RAS/MAPK and MiR-7	25880778
4-AP/TEA-sensitive K+ channel	Lung cancer	Gefitinib	K^+ channel inhibition potentiates Gefitinib efficiency via EGFR-Ras-Raf pathway modulation	28219421
**Magnesium channels**				
TRPM6/TRPM 7	Colon cancer	Doxorubicin	Low expression associated to resistance	26563869
Mrs2	Gastric cancer	Adriamycin	High expression associated to apoptosis resistance and cell proliferation	19242098
??	Myelogenous leukemia	Vincristine	Mg^{2+} needed for MDR-1 drug binding and extrusion	2900677
??	Ovarian and cervical cancer	Cisplatin	Mg^{2+} needed for PPM1D dependent drug-resistance	25154814
**Chloride channels**				
VRAC	Breast cancer	Doxorubicin	MDR1 expression regulates chloride current in resistant cells (reduced AVD)	16039989
	Epidermoïd cancer	Cisplatin	Reduced VRAC expression promotes resistance (reduced AVD)	17186499
	Lung adenocarcinoma	Cisplatin	Reduced VRAC expression promotes resistance (reduced AVD)	21454376
	Ehrlich ascites model	Cisplatin	Reduced VRAC expression promotes resistance (reduced AVD)	19846756
	Naspharyngeal carcinoma	Cisplatin	VRAC activity through P2Y pathway modulates sensitivity	25236172
ANO1	Breast cancer	Trastuzumab	Transcriptional regulation of HER2	27838298
	HNSCC[2]		ANO1 expression correlates with response to anti-EGFR therapy	25823819
CLIC1	Glioblastome	BCNU	Increased activity increases resistance	18590702
	Ovarian cancer		Increased expression increases resistance	27825122
	Choriocarcinoma	Methotrexate / Floxuridine	Increased expression associated to up-regulation of MRP1	27983917

ClC-3	Neuroendocrine tumor	Etoposide	Acidification of intracellular compartment induces drug chelation	17363491
	Erythroleukemia	Cisplatin	Acidification of intracellular compartment induces drug chelation	21538933
	Glioma	Cisplatin	Expression associated to cisplatin resistance	29963152
	Glioma	Cisplatin	Regulation of Akt and autophagy pathways	23408563
	Lung adenocarcinoma	Paclitaxel/Doxo rubicin	Up-regulation of NF-kB-signaling dependent P-gp expression	30230544
ClC-5	Myeloma	Bortezomib	ClC5 promotes prosurvival autophagy by inhibiting Akt-mTOR pathway	28899456
ANO1 / ClC3	Breast cancer		Chloride level impacts PI3K/Akt and STAT3 pathways to regulate HER2 transcription	29949674

[1] SCLC: Small Cell Lung Carcinoma
[2] HNSCC: Head and Neck Squamous Cell Carcinoma

© 2019 by the authors. Licensee MDPI, Basel, Switzerland. This article is an open access article distributed under the terms and conditions of the Creative Commons Attribution (CC BY) license (http://creativecommons.org/licenses/by/4.0/).
